# Neonatal imitation and early social experience predict gaze following abilities in infant monkeys

**DOI:** 10.1038/srep20233

**Published:** 2016-02-01

**Authors:** Elizabeth A. Simpson, Grace M. Miller, Pier F. Ferrari, Stephen J. Suomi, Annika Paukner

**Affiliations:** 1 Department of Psychology, University of Miami, Coral Gables, Florida, USA; 2 Dipartimento di Neuroscienze, Università di Parma, Parma, Italy; 3 Laboratory of Comparative Ethology, Eunice Kennedy Shriver National Institute of Child Health and Human Development, National Institutes of Health, Poolesville, Maryland, USA; 4 Clinical and School Psychology, University of Virginia, Charlottesville, Virginia, USA

## Abstract

Individuals vary in their social skills and motivation, the causes of which remain largely unknown. Here we investigated whether an individual’s propensity to interact with others measured within days after birth, and differences in infants’ early social environment, may predict a later social skill. Specifically, we tested whether neonatal imitation—newborns’ capacity to match modelled actions—and social experience in the first months of life predict gaze following (directing attention to locations where others look), in infant macaques (*Macaca mulatta*; *n* = 119). Facial gesture imitation in the first week of life predicted gaze following at 7 months of age. Imitators were better at gaze following than non-imitators, suggesting neonatal imitation may be an early marker predicting socio-cognitive functioning. In addition, infants with rich social environments outperformed infants with less socialization, suggesting early social experiences also support the development of infants’ gaze following competence. The present study offers compelling evidence that an individual difference present from birth predicts a functional social cognitive skill in later infancy. In addition, this foundational skill—gaze following—is plastic, and can be improved through social interactions, providing infants with a strong foundation for later social interaction and learning.

Social skills form the basis of the capacity to interact with others and to successfully integrate into society. Individual differences in adults’ social skill may be the result of two different yet interconnected processes: an individual’s natural potential to engage with others – related to individual differences in personality, intrinsic motivation, or genetic make-up; and the effect of the environment, either nurturing or suppressing this natural potential. While a retrospective analysis of the influences on social skills is valuable, prospective experimental studies of this issue can avoid sources of bias and confound. Here we investigated whether a newborn’s propensity to interact with others and the early social rearing environment predict a later socio-cognitive skill: gaze following (i.e., the ability to look where another individual is looking). We measured newborns’ social propensity with neonatal imitation (i.e., human and nonhuman primate (NHP) newborns’ ability to match modeled behaviours within days after birth^1^,^2^). We chose macaques for this study because humans and macaques exhibit similar social capacities across early infant development, including neonatal imitation and gaze following, with the added advantage that the rearing environment of macaques can be carefully controlled and manipulated.

Macaque newborns, like humans, engage in complex face-to-face interactions, including long bouts of mutual gaze^3^ and facial gesture imitation^2^,^4^. Both species exhibit striking individual differences in sociality from birth (for a review in humans, see^5^). For example, in humans and macaques, approximately half of newborns imitate and half do not^6^,^7^. While it is possible that this variability may be due to a transient cause, such as an infant’s state, a more intriguing possibility is that it may reflect a meaningful and stable individual difference. While this idea has been widely proposed^8^,^9^,^10^,^11^,^12^, it has yet to be thoroughly tested.

We hypothesized that individual differences in neonatal imitation may reflect individual differences in infants’ social cognitive skills, such as the ability to match another individual’s action with the infant’s own motor representation of that action. According to this hypothesis, observed actions activate one’s own action programs, thus facilitating action recognition, critical for early social interactions^11^. In monkeys, this system is functioning at birth^13^ and is expressed in neonatal imitation^11^. If this hypothesis is correct, neonatal imitation may positively predict later social skills^8^,^9^,^10^,^11^,^12^.

In support of this proposal, a handful of reports link neonatal imitation and other aspects of development (recent review:^14^). In humans, only one study examined neonatal imitation predictively and found it was associated with fewer looks away during an interaction at three months, potentially reflecting that imitators were more socially engaged^7^,^15^. In monkeys, neonatal imitators, compared to non-imitators, may better recognize social partners in the first week of life^16^ and exhibit more mature face viewing patterns at two to four weeks of age^17^. However, we know little about whether imitative skill predicts behaviour beyond the first month of life, or whether it predicts more advanced social skills.

One advanced social skill that emerges in the first year of life in human and nonhuman primates is the ability to follow another’s gaze into space^18^,^19^,^20^,^21^,^22^. Gaze following, like imitation, is a social skill that has been proposed to serve an important evolutionary function, allowing infants to use the gaze direction of older, more expert individuals to locate salient items, such as food, predators, and conspecifics^23^. By the middle of the first year of life, macaques follow the gaze of conspecifics^21^ and humans^22^, but their gaze following continues to improve into adulthood^24^,^25^,^26^, similar to humans (for a review:^20^).

Both neonatal imitation and gaze following require the interest and ability to track another individual’s behaviour^27^. In monkeys, neonatal imitators, compared to non-imitators, look more at the eye region of faces^17^, so imitators may be more likely to detect changes in such features. During face-to-face interactions, human and nonhuman primate newborns are sensitive to gaze engagement (e.g.^28^), a powerful cue for the development of social skills^29^. This link between early social skills and sensitivity to gaze may remain stable during development; however, the extent to which these skills are directly associated with one another remains untested.

As in neonatal imitation, there are interindividual differences in gaze following^30^. These individual differences may also be, in part, due to differences in infants’ early social experiences. In social species, including macaques and humans, the early social environment appears critical in the development of social skills^31^,^32^,^33^. While there is evidence of rudimentary gaze following in human newborns^34^, it continues to mature in the first year of life, during which time it may be influenced by social experience, such as through reinforcement learning^35^,^36^. That is, a rich social environment—especially one with joint attention interactions—provides opportunities for infants to learn links between others’ gaze and relevant environmental stimuli^37^,^38^. This hypothesis, however, is difficult to test in humans, as we have limited control over infants’ early social environments.

In the present study, our first goal was to explore whether imitation in the first week of life contributes to the development of a later social skill—gaze following—at 7 months, in infant macaques reared under controlled environmental conditions. Based on reports that individual differences in neonatal imitation may be associated with later social skills (e.g.^17^), we predicted that neonatal imitators would be more advanced in their gaze following behaviour than non-imitators. Our second goal was to explore whether early social experiences influence social skill development. To assess this, we compared infants with varying levels of social experience—high-socialization infants, housed with three to four of their peers—with low-socialization infants, housed individually with more limited peer interactions. We designed these environmental manipulations to mimic the variability in natural early social environments, with some infants receiving more opportunities for social interactions than others. We hypothesized that neonatal imitators, compared to non-imitators, would exhibit better gaze following due to their greater interest or skill in social interactions. We also hypothesized that high-socialization infants, compared to low-socialization infants, would exhibit better gaze following due to their increased exposure to social cues, enhancing their interest or skill in social interactions. Finally, we predicted imitation and social experience may interact, in one of two ways: imitators, who may be more socially motivated^39^, may show greater benefits of socially enriched early environments, compared to non-imitators, and therefore may better follow gaze. Alternatively, non-imitators, who may be initially less intrinsically social^17^, may benefit more from socially enriched early environments compared to imitators, and therefore may exhibit greater rearing-related improvements in gaze following.

## Results

There was interindividual variability in neonatal imitation (see Supplemental Materials, Fig. S1). In the gaze following task, we analysed the proportion of infants’ correct responses against chance (0.50). Data were normally distributed with no outliers. All *t* tests were two-tailed and included Bonferroni corrections. We confirmed, with one-sample *t* tests, that infants followed gaze above chance for both head trials (*M* = 0.61, *SD* = 0.15), *t*(118) = 8.52, *p* < 0.001, *d* = 0.78, and head + torso trials (*M* = 0.66, *SD* = 0.15), *t*(118) = 11.59, *p* < 0.001, *d* = 1.06. For each sub-group of infants (imitators and non-imitators within both high- and low-socialization rearing), gaze following was also above chance, *p*s < 0.01, Supplemental Table 1. Out of 119 infants, 105 (82% of low-socialization infants and 89% of high-socialization infants) performed gaze following at rates above chance.

**Figure 1:**
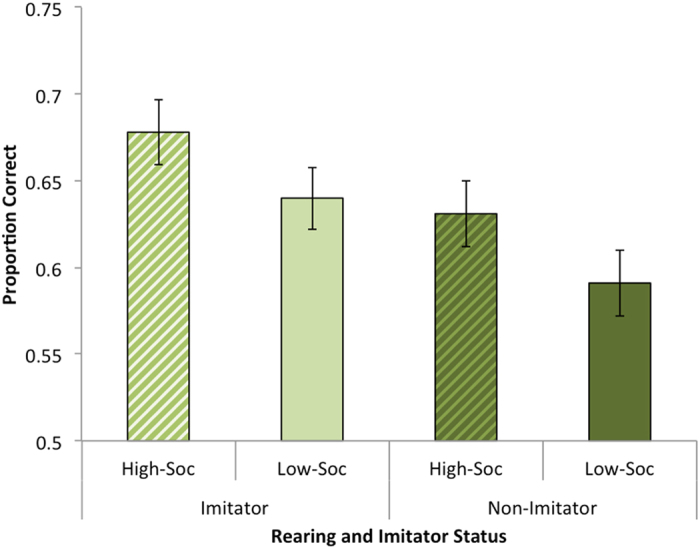
Proportion of correct gaze following responses (chance = 0.50) for main effects of Rearing—high-socialization (striped bars) and low-socialization (solid bars) and Imitator status—imitator (light bars) and non-imitator (dark bars). Error bars reflect standard error of the mean.

We tested our hypothesis that interindividual differences in gaze following would be predicted by neonatal imitation and rearing with a 2 × 2 × 2 mixed-design ANOVA with the within-subjects variable Trial type (head + torso-turn, head-turn), and the between-subjects variables Rearing (high- and low-socialization) and Imitator status (imitator, non-imitator). There was a main effect of Trial type, with a greater proportion of correct responses in head + torso trials (*M* = 0.66, *SD* = 0.15) compared to head only trials (*M* = 0.62, *SD* = 0.15), *F*(1,115) = 4.66, *p* = 0.033, η_p_^2^ = 0.04. There was a main effect of Imitator status, with imitators exhibiting a greater proportion of correct responses (*M* = 0.66, *SD* = 0.10) compared to non-imitators (*M* = 0.61, *SD* = 0.11), *F*(1,115) = 6.68, *p* = 0.011, η^2^ = 0.06, Fig. 1. There was a main effect of Rearing, with a greater proportion of correct responses by high-socialization (*M* = 0.66, *SD* = 0.09) compared to low-socialization infants (*M* = 0.62, *SD* = 0.11), *F*(1,115) = 4.39, *p* = 0.038, η^2^ = 0.04, Fig. 1. There were no interactions, *p*s > 0.05.

## Discussion

We found support for our prediction that macaque neonates’ imitative capacity—to match facial gestures produced by a model—assessed in the first week of life, predicts a social skill in later infancy—gaze following at 7 months. The present study offers the first evidence (in any species, including humans), to our knowledge, that an individual difference present from birth modulates a social cognitive skill in later infancy. Neonatal imitation may, indeed, reflect a meaningful individual difference, as previously hypothesized^8^,^9^,^10^,^11^,^12^. This finding corroborates reports of higher sensitivity and responsivity to social cues in monkey neonatal lipsmacking (LPS; an affiliative facial gesture involving rapid opening and closing of the mouth) imitators, compared to non-imitators, who, in the first week of life may also better recognize social partners^16^, and, at one month, may attend more to the eye region of faces^17^. This finding is consistent with a report that, in human infants, neonatal imitators exhibit fewer looks away during a face-to-face interaction at 3 months^8^,^15^, perhaps because imitators were more socially engaged. However, the present findings are the first that suggest that imitators, compared to non-imitators, may possess more mature or functional social skills.

From a neurobiological perspective, imitative skills and joint attention activate different brain networks. The former relies on neural mechanisms mapping others’ actions (e.g., gestures) onto their own motor representation of that action, named the mirror mechanism^13^. The latter likely represents a building block for the development of more sophisticated mentalizing capacities (i.e., theory of mind) that, in adults, involves a network including the temporo-parietal junction and medial prefrontal cortex^40^. Although each system processes a different type of social information, both are part of the social brain. The mirror mechanism has been well described in monkeys and humans^41^. This mechanism emerges early in development and infants’ early capacity to imitate facial gestures probably relies upon it^4^,^11^,^13^. In fact, these systems may perform complementary, non-overlapping functions in service of social cognition^42^, acting together to support action understanding. For example, both the mirror mechanisms and the mentalizing system are engaged during joint actions^43^, while viewing or imagining social interactions^44^,^45^, and while viewing communicative gestures^46^.

The present study has implications for the development of social skills in human infants. Unlike in humans, we can experimentally manipulate the timing and nature of infant macaques’ early social experiences. In doing so, we found support for our prediction that infants reared in a high-socialization environment outperformed infants reared in a low-socialization environment. While both groups performed above chance, there were significant individual differences accounted for by infants’ early social environments. Our findings are consistent with the hypothesis that gaze following is learned through social exposure^25^,^38^ and that, in macaques, early social experiences may affect social skills^4^,^33^,^47^. On the one hand, this result is promising because it suggests there is some plasticity in this social cognitive skill. Particularly, in infants who show early deficits, there may be ways of supporting the development of this skill by providing them with additional social interaction opportunities. While we predicted that early imitative capacity might interact with early rearing, we instead found that all infants appeared to benefit from peer socialization, regardless of their initial imitative skill.

Notably, even infants who were non-imitators and did not receive enriched social interactions with peers—the most “at risk” group—nonetheless performed gaze following at above-chance levels. Of course, infants in the present study all had some peer and human caregiver social interactions, even if lower than in naturalistic contexts, which may have been sufficient for healthy social development. In contrast, infants raised in an environment with even less social stimulation may display insufficient or delayed gaze following due to their limited exposure to social stimuli, which may have downstream consequences given that in humans, gaze following is foundational for higher-level social development (e.g., joint attention;^23^,^26^, theory of mind^48^,^49^, social learning^50^). Through studies such as this one we can begin to understand the interdependence of different skills.

Many questions remain. The present study does not allow us to determine the precise factors within the social environment that may be supporting infants’ gaze following skills. For example, through reinforcement learning^37^ infants in the high-socialization condition had more opportunities to learn that gaze following provided useful information about their environment. In addition, early socialization may have altered infants’ social motivations^38^. If so, infants in the high-socialization condition, who had greater social experiences compared to the low-socialization infants, may have been more motivated to interact with social partners because they found such interactions more rewarding. In theory, social skill and intrinsic social motivation may influence one another bi-directionally, a challenge outside the scope of the present study, but perhaps relevant to understanding autism spectrum disorders (ASD)^51^.

Finally, the present study is limited in that we cannot determine the specific aspects of the model—movement of the eyes or head alone—that infants used to follow others’ gaze. In the present study, our models moved both their eyes and head together because, while adult monkeys follow gaze cues that include just the eyes, juvenile monkeys do not^26^. In addition, gaze following performance may have been better if the stimulus had been provided by a conspecific; however, this is not feasible to test with a live adult monkey model and previous studies have already demonstrated that orienting stimuli provided by a human experimenter are effective in triggering gaze shift responses in juvenile and adult monkeys^26^.

Social skills are foundational for successfully integrating into society, yet we still know little about the causes of individual differences in social skills^52^. In a prospective experiment with infant monkeys we explored the contributions of an individual’s natural potential to engage with others and the effect of the environment, nurturing or suppressing this potential. We found a positive association between infants’ neonatal imitation in the first week of life and gaze following ability at 7 months of age. This finding suggests that neonatal imitation assessments might compliment other screening tools for identifying infants at heightened risk for impaired social function^17^. At the same time, we found evidence that gaze following skills are plastic, positively influenced by early social experiences. This finding has clinical implications for populations at-risk for disorders, such as ASD, characterized by deficits in both imitation and gaze processing^30^,^53^,^54^,^55^,^56^. While we know of no published attempts to improve gaze following in high-risk infants, our findings suggest that such interventions might be worthwhile. Finally, we found evidence that the development of gaze following is resilient, developing even with limited opportunities for social interaction, and even among infants who exhibit low rates of neonatal imitation. In sum, the present findings provide support for the hypothesis that individual differences in neonatal imitation may reflect infants’ social cognitive skills, highlighting the importance of continued investigation into both early screening and potential interventions for at-risk infant populations.

## Method

### Subjects

Infant rhesus macaques (*Macaca mulatta*) participated in the neonatal imitation assessment between 1–8 days of age and in a gaze following assessment at approximately 7 months of age (*M* = 234 days, *SD* = 15). Subjects included singly housed surrogate-reared, low-socialization infants (*n* = 61; 28 females), and peer-reared, high-socialization infants (*n* = 58, 23 females). On the day of birth, infants were separated from their mothers and raised in a primate nursery. Infants were raised identically for the first five weeks. Once the youngest infant reached 37 days of age, infants were placed into groups. High-socialization infants were raised in groups of three to five peers. Low-socialization infants were individually housed, assigned to playgroups composed of three to four peers housed together two hours a day, five days a week. See Supplemental Materials for details.

### Materials and Procedures

#### Neonatal Imitation Test

We tested infants three times a day, every other day, in the first week of life. Infants viewed live stimuli, including a lipsmacking gesture (LPS; rapid opening and closing of the mouth) and a control (CTRL) condition, consisting of a 15-cm diameter striped Disk, rotated back and forth 180°. Condition order was randomized between subjects. Each session began with a 40-second static baseline in which the monkey was faced by a human experimenter presenting a still face in the LPS condition and a still disk in the control condition. This baseline was followed by a 100-second stimulus period consisting of a 20-second dynamic stimulus presentation and a 20-second static period (still stimulus), repeated 3 times: dynamic-static-dynamic-static-dynamic. Sessions were videotaped and experimenters blind to the experimental condition coded facial gestures, offline. Infants were classified as imitators if they produced an increase in LPS (rate per sec) from the baseline (still face) to the stimulus period (LPS face) in the LPS condition (matching the model), to a greater extent than the increase in LPS from the baseline (still disk) to the stimulus period (disk rotating) in the control condition, averaged across days. Using this classification, 61 infants were imitators (29 high-socialization, 32 low-socialization) and 58 infants were non-imitators (29 high-socialization, 29 low-socialization). See Supplemental Materials for details.

#### Gaze Following Test

Using an experimental design adapted from^30^, infants were tested over four successive days, receiving 10 trials per day, for a total of 40 trials. A familiar caretaker handled the infants. An actor sat approximately two feet in front of the infant, and two evaluators sat approximately four feet behind the actor at opposite 45° angles. Thus, one evaluator was slightly to the left of the actor, and the other was slightly to the right of the actor. The actor sat at eye level with the infant and engaged in various attention getting behaviours to facilitate eye contact. Upon making eye contact, the actor looked either right or left, moving either the head or head + torso 90°, consistent with the direction of gaze, and held this position for approximately five seconds. Thus, there were four possible movements for the infant to observe: head left, head + torso left, head right, head + torso right. The direction of the actor’s eye gaze shift, and movement of torso or head were counterbalanced so the infant saw 10 of each. Head + torso trials contained larger and more obvious movement cues, while head-only trials were thought to be more challenging as they involved a subtler cue. Prior to the test session, one evaluator was assigned to call out the direction of the infant’s first gaze shift, after the actor’s movement. If a monkey did not shift his or her eyes for five seconds, then the eye movement was recorded as “straight ahead.” To ensure accuracy, the second evaluator either agreed or disagreed with the first evaluator’s statement of gaze direction. Upon disagreements or instances of the infant failing to attend to the actor’s movement, the trial was repeated until the evaluators agreed. We assessed infants’ performance by the proportion of correct responses—looking left or right, consistent with the model—out of the total number of left and right responses^37^,^57^,^58^.

## Ethics Statement

This study was carried out in accordance with the recommendations in the Guide for the Care and Use of Laboratory Animals and complied with the Animal Welfare Act. The *Eunice Kennedy Shriver* National Institute of Child Health and Development’s Animal Care and Use Committee approved this study.

## Additional Information

**How to cite this article**: Simpson, E. A. *et al.* Neonatal imitation and early social experience predict gaze following abilities in infant monkeys. *Sci. Rep.* **6**, 20233; doi: 10.1038/srep20233 (2016).

## Supplementary Material

Supplementary Information
