# Supplementary material for: Neonatal imitation and early social experience predict gaze following abilities in infant monkeys
Source: Sci Rep. 2016 Feb 1;6:20233. doi: 10.1038/srep20233 (PMC4735292; doi:10.1038/srep20233)
Supplement: Supplementary Information [file srep20233-s1.pdf]

Neonatal imitation and early social experience predict gaze following abilities in infant monkeys

Supplemental Materials

Elizabeth A. Simpson[#], Grace M. Miller[#], Pier F. Ferrari, Stephen J. Suomi, and Annika Paukner*

[#]Authors contributed equally.

*Corresponding author.

Method

Subjects

On the day of birth, infants were separated from their mothers and raised in a primate nursery. For the first 14 days, infants were housed in a room containing 5-10 incubators. Infants were individually reared in plastic-topped incubators (51 × 63.8 × 64.3 cm) maintained at 24-28°C. Infants could see and hear other infants, but remained physically separated. Incubators contained one soft toy, two hard toys, three fleece blankets, and a surrogate made from an upright, flexible polypropylene cylinder covered in fleece. A two-ounce bottle containing Similac formula was attached to the surrogate for the first week of life. Toys and fleece blankets were replaced daily. At eight days of age, the plastic incubator top was replaced with a wire top, and the polypropylene cylinder surrogate was replaced with a hanging, fleece-covered surrogate pouch, into which the infant could climb. Two-ounce bottles of formula were attached to the side of the cage. Additionally, cages contained four hanging enrichment toys, two hard toys, and three blankets. Hard toys and fleece blankets were replaced daily. At fifteen days of age, infants were moved from the incubator room into an adjacent room lined with 61 × 61 × 76 cm cages. Infants were placed into one of the cages, along with three blankets, three floor toys, two hanging toys, and a hanging surrogate. The surrogates were changed weekly, and enrichment toys were rotated daily. At fifteen days of age infants also began receiving six minutes of handling by a member of the nursery staff on weekday mornings. The monkeys were rotated so that staff members did not hold the same monkey more than once every four days. On day sixteen infants began drinking formula through 500 ml bottles with metal sipper tube.

Infants were assigned to rearing conditions on their day of birth. Peer-reared (high-socialization) infants were raised in groups of three to five similar-aged peers in a 71 × 81 × 152 cm cage. High-socialization infants were placed into their peer groups when the youngest member of the group reached 37 days of age. Until then they remained in their individual cages. Each peer cage contained three floor toys, one fleece blanket, and one hanging surrogate per infant, in addition to two hanging toys. When each infant in the peer group turned four months of age, the single hanging surrogate was removed from the cage. Thus,

when the youngest infant turned four months of age, no surrogates remained in the cage. Infants remained in their peer groups until they were weaned around seven to eight months of age (after gaze following testing was completed).

Unlike peer-reared infants, surrogate-reared (low-socialization) infants were individually housed in 61 × 61 × 76 cm cages throughout their stay in the nursery. Cages contained three fleece blankets, three floor enrichment toys, two hanging enrichment toys, and a hanging surrogate. Low-socialization reared infants had access to their surrogate throughout their stay in the nursery. To facilitate socialization, low-socialization infants were assigned to playgroups composed of three to four similar-aged infants. Once the youngest member of the playgroup reached 37 days of age, infants in were placed into a 71 × 81 × 152 cm cage with their playgroup for two hours a day, five days a week. Only half of the play cage was accessible to the infants until the youngest infant turned two months old. The play cage contained enrichment toys, one hanging surrogate, three to four fleece blankets, and one water bottle. Once the youngest infant turned two months old, the entire cage was made accessible, and another hanging surrogate and water bottle was added to the cage.

For the first thirty days of life, infants were fed bottles of Similac formula every two hours from 8am to 8pm. After 30 days, infants were fed at 8am and 4pm, Monday through Friday, and at 7:30am and 1:30pm on weekends. The amount of Similac provided to infants varied by infants' age and weight. At 16 days of age, Purina LabDiet 5045 High Protein Monkey Diet chow was introduced to their diet, in addition to Similac. The feeding regimen eventually transitioned from a Similac-only diet, to a chow-only diet by six months of age. Infants also received a daily afternoon snack of nuts, fruit, grains, or seeds beginning at two months of age. Water was available ad libitum from a lixet system. In the incubator room and the large cage room, lights were on from 7:00 to 21:00.

Details of the Neonatal Imitation Procedure and Classification

Infants were tested three times a day, every other day, in the first week of life (days 1-2, 3-4, 5-6, and 7-8), for up to four days. There was at least an hour between each test session. A demonstrator presented infants with two stimuli, one during each session, at a distance of

approximately 30 cm at eye-level with the infant: a lipsmacking gesture (rapid opening and closing of the mouth) and a nonsocial control condition (a white plastic disk with orthogonal stripes—which were either black/red or green/yellow—slowly rotated clockwise and counter-clockwise). Each stimulus type was presented once a day to infants; the order of stimulus presentations remained the same for each infant but was randomized between infants. In each test session, one experimenter held the infant, a second experimenter—the demonstrator—served as the source of the stimuli, and a third experimenter was the time-keeper who ensured stimuli were presented for appropriate lengths. All sessions were videotaped. Individual demonstrators were randomly assigned to conditions but remained consistent across days within each infant.

At the beginning of a trial, a 40 sec baseline was conducted, in which the demonstrator displayed a calm, neutral facial expression (or the still disk in control). The demonstrator then displayed a facial movement (lipsmacking) or rotated the disk for 20 seconds, followed by a still/neutral facial expression (still disk in control) period for 20 sec. This movement-still face sequence was repeated three times.

Infants' mouth movements were coded off-line, frame-by-frame (30 frames per second) from video. Lipsmacking was operationally defined as a high frequency opening and closing of the mouth without sound production in which the lips were required to part and then rejoin within 2 seconds. Observers were blind to the stimulus. Inter-observer agreement was high, $r = .950$, $p < .001$, $n = 66$.

Categorical Classification: Imitators and Non-Imitators. Lipsmacking gestures rates (per 60 sec) from Baseline and Stimulus were averaged across all test days in each condition. Infants were classified as imitators and non-imitators. Infants were LPS imitators if they produced an increase in LPS from the baseline to the stimulus period in the LPS condition (matching the model), to a greater extent than the increase in LPS from the baseline to the stimulus period in the control condition, averaged across days [1-3].

Continuous Classification: Imitation Index. We computed a LPS Imitation Index (imitation strength score), using the average gesture rates across days, while considering both baseline response rates and responses to the Control (CTRL) condition. We used the formula: LPS Imitation Index = $[(LPS_{Stimulus} – LPS_{Baseline})_{LPS\ Cond}] - [(LPS_{Stimulus} – LPS_{Baseline})_{CTRL\ Cond}]$. We calculated a difference score: LPS produced in Stimulus and subtracted from it LPS produced in Baseline (Still-Face). This difference score was computed for the LPS and CTRL conditions, and we subtracted the CTRL condition from the LPS condition to obtain the difference of the difference scores. The resulting value is small or negative if there was a greater imitative response in the CTRL condition (non-Imitators), and greater than zero if there was a greater imitative response in the LPS condition (imitators). For further classification details, see [3].

Results and Discussion

Preliminary Neonatal Imitation Analyses

We first explored interindividual variability in neonatal imitation. Imitators produced a larger increase in gestures, in the LPS condition, from the baseline (M = 1.91, SD = 1.91) to the stimulus period (M = 5.1, SD = 3.8), compared to the CTRL condition increase from the baseline (M = 3.6, SD = 2.6) to the stimulus period (M = 3.7, SD = 2.6), $t(60) = 8.58$, $p < .001$, $d = 1.10$, whereas non-imitators exhibited the opposite pattern, producing a greater increase in LPS, in the CTRL condition, from the baseline (M = 2.4, SD = 2.2) to the stimulus period (M = 4.2, SD = 3.0), compared to in the LPS condition, from the baseline (M = 3.9, SD = 3.4) to the stimulus period (M = 3.6, SD = 3.0), $t(61) = 7.58$, $p < .001$, $d = .96$. In addition to the categorical classification of infants as imitators and non-imitators, we also calculated a LPS imitation index score for each infant, which revealed varying levels of imitative capacity, even within the imitators, Figure S1. We obtained the same pattern of results when imitation was a continuous variable (see Fig. S2); therefore, for simplicity, we hereafter use the categorical classification, i.e., labeling infants imitators and non-imitators, consistent with previous approaches [e.g., 1,2,5,6]. Our data are consistent with previous reports in newborn humans and macaques, suggesting that infants vary in their imitative capacity, with approximately half of infants

imitating consistently, while approximately half do not imitate [1,4]. In sum, these results lend support for the proposal that neonatal imitation is a good skill to assess in terms of interindividual variability.

Preliminary Gaze Following Analyses

We excluded 24% of trials because infants failed to produce a usable response (e.g., looked up or straight-ahead). This resulted in the exclusion of an equal proportion of trials for imitators and non-imitators, $t(117) = .58$, $p = .57$, but a higher proportion of trials excluded for low-socialization ($M = .27$, $SD = .16$) compared to high-socialization infants ($M = .22$, $SD = .14$), $t(117) = 2.00$, $p = .048$, $d = .37$. Preliminary analyses on the proportion of correct responses revealed no effects of sex (head: $t(117) = .32$, $p = .75$; head+torso: $t(117) = .18$, $p = .86$), or age (head: $r = -.13$, $p = .17$; head+torso: $r = -.14$, $p = .14$).

Consistency of Imitation and Gaze Following Responses

We carried out a repeated measures ANOVA on the neonatal imitation index with the within subjects factor of test session (1, 3, 5, 7 days of age). There was no significant change in imitation across days, $F(3,333) = .52$, $p = .672$. We carried out a repeated measures ANOVA on gaze-following scores with the within subjects factor of test session (1, 2, 3, 4). There was no significant change in gaze-following across sessions, $F(3,333) = 1.03$, $p = .378$. Together, these results suggest that there were no detectable systematic changes in infants' performance across sessions in either task.

Supplemental References

1. Ferrari, P. F., et al. Interindividual differences in neonatal imitation and the development of action chains in rhesus macaques. *Child Dev.* **80**, 1057-1068 (2009).
2. Paukner, A., Simpson, E. A., Ferrari, P. F., Mrozek, T. & Suomi, S. J. Neonatal imitation predicts how infants engage with faces. *Dev. Sci.* **17**, 833-840 (2014).
3. Simpson, E. A., Paukner, A., Sclafani, V., Suomi, S. J. & Ferrari, P. F. Lipsmacking imitation skill in newborn macaques is predictive of social partner discrimination. *PLOS ONE* **8**, e82921 (2013).
4. Heimann, M. Notes on individual differences and the assumed elusiveness of neonatal Imitation in *The imitative mind: development, evolution, and brain bases* (eds Meltzoff, A. N. & Prinz, W.) 74-84 (Cambridge University Press, 2002).
5. Paukner, A., Ferrari, P. F. & Suomi, S. J. Delayed imitation of lipsmacking gestures by infant rhesus macaques (*Macaca mulatta*). *PLoS ONE* **6**, e28848-e28848. (2011).
6. Simpson, E. A., Paukner, A., Suomi, S. J. & Ferrari, P. F. Visual attention during neonatal imitation in newborn macaque monkeys. *Dev. Psychobiol*. **56**, 864-870. (2014).

Supplemental Figure

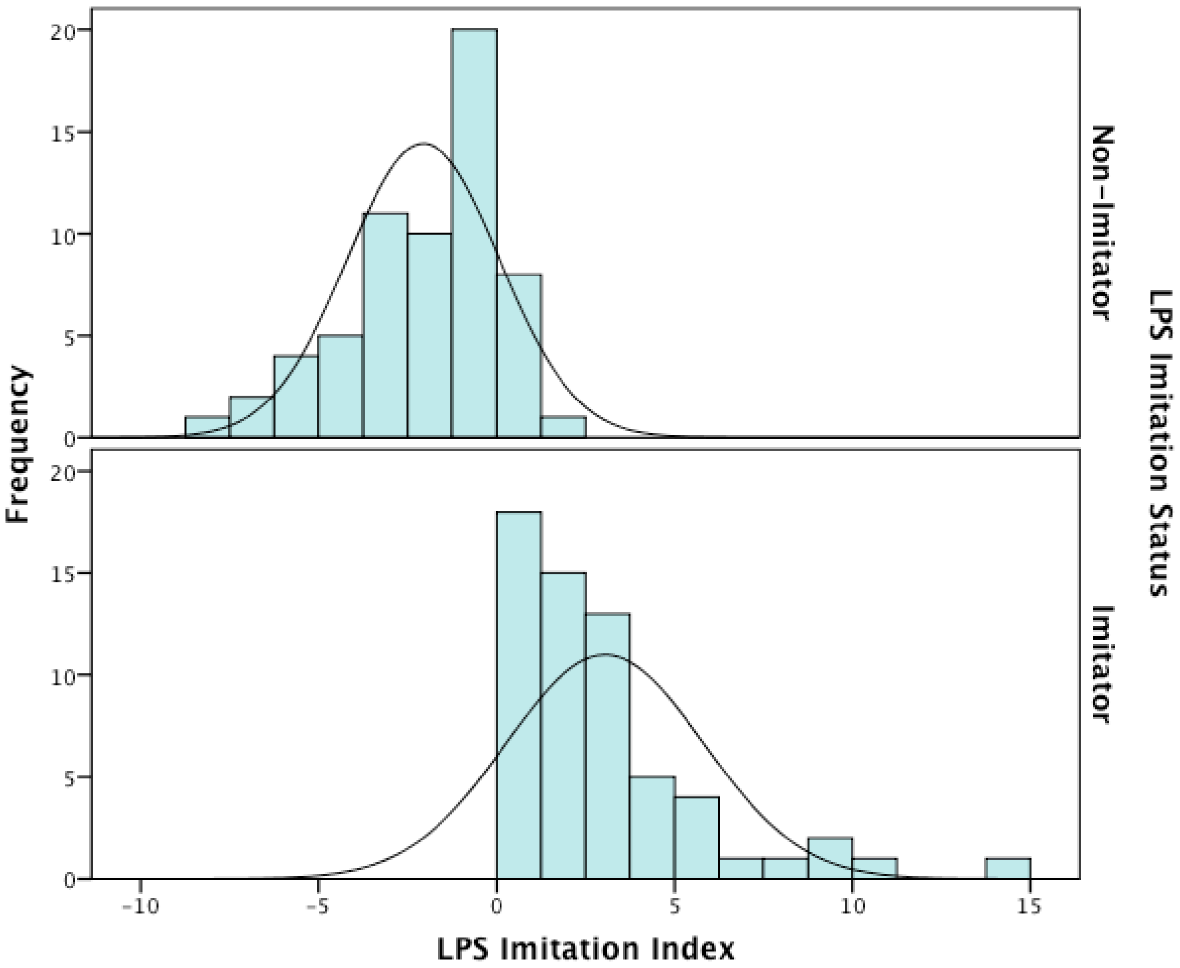

Figure S1. Frequency of infants with various rates of lipsmack (LPS) imitation. LPS Imitation Index = $[(LPS_{Stimulus} - LPS_{Baseline})_{LPS\ Cond}] - [(LPS_{Stimulus} - LPS_{Baseline})_{CTRL\ Cond}]$. The resulting value is small or negative if there was a greater imitative response in the CTRL condition (top graph; Non-Imitators), and greater than zero if there was a greater imitative response in the LPS condition (bottom graph; Imitators). This shows the variability in LPS matching responses, with some infants exhibiting a high frequency of matches (far right) and some exhibiting few or no matches (far left).

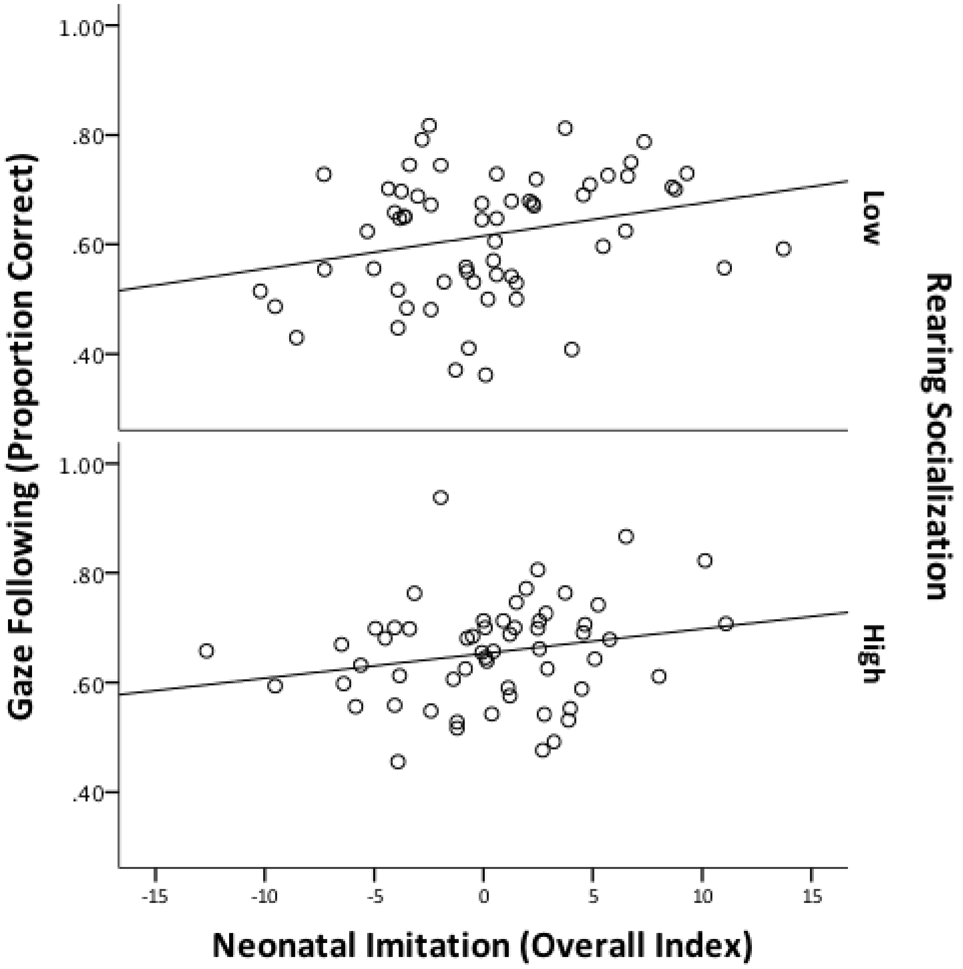

Figure S2. Both low-socialization (top graph) and high-socialization (bottom graph) rearing groups showed a positive relationship between neonatal imitation and gaze following. Infants' overall imitation index predicted their gaze following, $F(1,116) = 5.76$, $p = .004$, with a correlation coefficient of .30, indicating that approximately 9% of the variability in gaze following scores can be accounted for by infants' imitative capacity and early rearing environment.

Supplemental Table

Status	Rearing	Condition	*M*	*SD*	*t*	*df*	*p*	*d*
Imitator	High-Socialization	Head	0.67	0.09	9.81	28	< 0.001	1.82
		Torso	0.69	0.12	8.19	28	< 0.001	1.52
	Low-Socialization	Head	0.58	0.13	3.27	30	0.003	0.59
		Torso	0.69	0.15	7.08	30	< 0.001	1.27
Non-Imitator	High-Socialization	Head	0.61	0.14	4.44	28	< 0.001	0.82
		Torso	0.65	0.15	5.16	28	< 0.001	0.96
	Low-Socialization	Head	0.60	0.19	2.79	29	0.009	0.51
		Torso	0.60	0.14	3.70	29	0.001	0.67

Table S1. One-sample *t* tests comparing gaze following performance to chance (.50) for each group of infants (imitators and non-imitators, high- and low-socialization infants) within each condition (head-turn and head+torso-turn). Means (*M*), standard deviations (*SD*), and effect sizes (Cohen's *d*) are also reported.
